# Bainitic Ferrite Plate Thickness Evolution in Two Nanostructured Steels

**DOI:** 10.3390/ma14154347

**Published:** 2021-08-03

**Authors:** Victor Ruiz-Jimenez, Jose A. Jimenez, Francisca G. Caballero, Carlos Garcia-Mateo

**Affiliations:** MATERALIA Research Group, Department of Physical Metallurgy, National Center for Metallurgical Research (CENIM-CSIC), Avenida Gregorio del Amo, 8, 28040 Madrid, Spain; vicruizj@gmail.com (V.R.-J.); jimenez@cenim.csic.es (J.A.J.); fgc@cenim.csic.es (F.G.C.)

**Keywords:** bainite, transformation kinetics, retained austenite, plate thickness

## Abstract

Bainitic ferrite plate thickness evolution during isothermal transformation was followed at the same holding temperatures in two nanostructured steels containing (in wt.%) 1C-2Si and 0.4C-3Si. A dynamic picture of how the bainitic transformation evolves was obtained from the characterization of the microstructure present at room temperature after full and partial transformation at 300 and 350 °C. The continuous change during transformation of relevant parameters influencing the final scale of the microstructure, YS of austenite, driving force of the transformation and evolution of the transformation rate has been tracked, and these variations have been correlated to the evolution of the bainitic ferrite plate. Instead of the expected refinement of the plate predicted by existing theory and models, this study revealed a thickening of the bainitic ferrite plate thickness as the transformation progresses, which is partially explained by changes in the transformation rate through the whole decomposition of austenite into bainitic ferrite.

## 1. Introduction

Bainitic transformation on silicon-rich steels with medium and high carbon content can take place at homologous temperatures as low as T/Tm ~0.25, where Tm is the absolute melting temperature. The displacive and diffusionless nature of bainitic transformation is exploited so the final microstructure is an intimate mixture of nanoplates of bainitic ferrite (30–80 nm) interwoven with thin films of carbon-enriched retained austenite. As bainitic ferrite plate thickness essentially characterizes the mean free slip distance [1,2], the nanoscale of the achieved microstructure is mainly responsible for its high hardness (650–700 HV), ultrahigh strength (2–2.3 GPa) and good toughness (30 MPa m^1/2^) [3,4,5,6,7].

Some of the parameters controlling the final scale of the bainitic ferrite plates have been already identified [1,8,9,10,11,12], and the decrease in the scale of the bainitic ferrite plates as transformation temperature T_Iso_ decreases has been correlated to the increase in the yield strength of the parent austenite ($YS_{\gamma }$), the driving force for the transformation (∆G) and the dislocation density (ρ). Strong austenite or a large driving force results in finer plates, the former because there is a larger resistance to interface motion and the latter because an increased nucleation rate leads to microstructural refinement by impingement between adjacent plates. 

As in the case of martensite, bainite growth causes strain, and that strain is plastically accommodated, generating in the austenite adjacent to the bainite plates dislocation debris (ρ) that also resists the advance of the bainite/austenite interface; note that the magnitude of ρ increases inversely to the transformation temperature (T_Iso_) [1].

Such studies offer a static picture in the sense that the chemical composition of the bulk and transformation temperature T_Iso_ are used to calculate the magnitudes of the $YS_{\gamma }$ and ∆G that finally lead to an estimation of the plate thickness at the end of the transformation [9,10,11,12,13].

Keeping in mind that during transformation at a given T_Iso_ there is a continuous variation of the ∆G and $YS_{\gamma }$ values, it should be possible to extract a more dynamic picture of the system evolution. However, in the above-mentioned literature, there is not, to the authors’ knowledge, a single reference describing the evolution of the bainitic ferrite plate thickness as the transformation progresses. With this purpose, in this work, a methodology is adopted where a combinational study using high-resolution dilatometry, X-ray diffraction (XRD) and SEM allows us to extract the necessary information from the microstructure at room temperature to obtain a dynamic picture of the transformation and microstructure evolution at a given temperature T_Iso_. The results will show that there is a strong correlation between the rate of the transformation and how the plate thickness evolves. The sluggishness of the transformation in the last stages and the unexpected plate thickness evolution as the transformation advances are rationalized on the basis of the obtained results, stressing the importance of considering the dynamic character of the evolving microstructure and the influence that it has on the final microstructure.

## 2. Materials and Methods

A high-carbon designed steel and a medium-carbon commercial steel (SCM40), both containing more than 1.5 mass% Si to prevent carbide precipitation from austenite during the bainitic transformation [14], have been used for the present work. These materials, named 1C2Si and 04C3Si, respectively, were produced by Sidenor (Basauri, Spain), and their nominal chemical compositions can be found in Table 1. They have been selected because, as it will be shown, although they exhibit very different transformation kinetics, nanobainitic microstructures can be developed in both materials.

Thermal treatments were performed in a Bahr 805A (TA Instruments, Hüllhorst, Germany) high-resolution dilatometer, which enables the study of phase transformations by monitoring the changes in the sample length. The temperature can be increased by an induction heating coil, whereas it can be decreased by using helium as quenching gas. Temperature is controlled by a K-type thermocouple welded to the central part of the sample surface. The dilatometry tests were performed using fused silica push-rods to measure longitudinal length changes. Cylindrical specimens of 10 mm length and 4 mm diameter were used.

**Figure 1:**
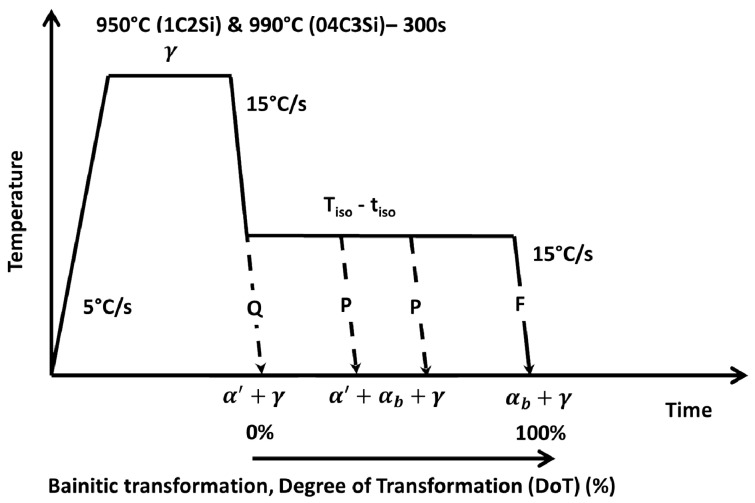
Scheme of the heat treatments applied in this work. Q stands for quench; P and F stand for partial and full transformation, respectively. The expected phases at relevant stages of the treatment are also indicated, where $\gamma ,\;\alpha_{b}$ and $\alpha^{\prime }$ stand for austenite, bainitic ferrite and martensite, respectively.

**Figure 2:**
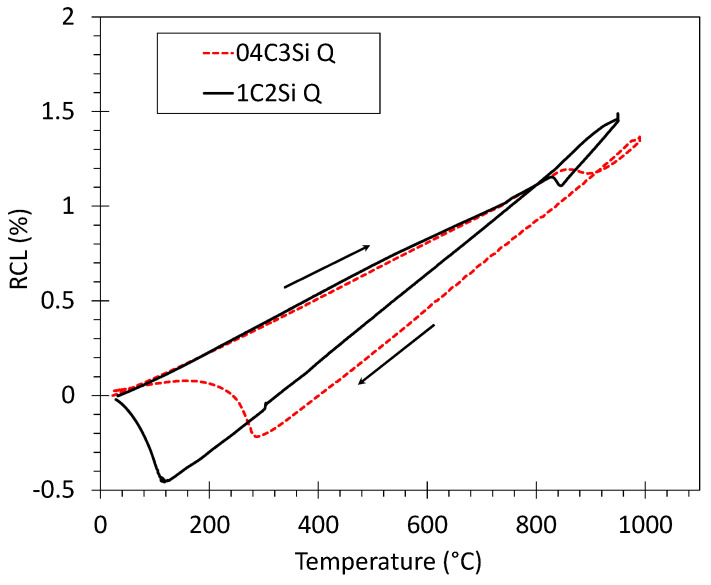
Relative change in length (RCL) as a function of T during quench (Q) experiments consisting of heating up to selected austenitization temperature at 5 °C/s and subsequent cooling down to room temperature at 15 °C/s.

In order to establish the heat treatment conditions, so bainitic transformation takes place at selected isothermal transformation temperatures, T_Iso_, from a fully austenitized condition, samples from both alloys were heated at 5 °C/s to a temperature of 950 °C for 1C2Si and 990 °C for 04C3Si, held for 5 min and cooled down to room temperature at a rate of 15 °C/s (treatment Q in Figure 1). From the relative change in length (RCL) vs. temperature curves (Figure 2) it was clear that selected austenitization T and cooling rate are sufficient to allow full austenitization of the as-received microstructure and to avoid ferrite–pearlite transformation on cooling to room temperature, as the only transformation detected is martensite. Determination of Ac1, Ac3, Acm and Ms (martensitic transformation start) temperatures from dilatometric curves was completed following the procedures described in [17,18], and the results are reported in Table 1. The same table also presents the Ms and Bs (bainitic transformation start temperature) calculated according to [15] and [16], respectively, and the relevance of such calculations will become clear later in the text. Based on those results, for the bainitic isothermal treatments, the chosen T_Iso_ (Ms < T_Iso_ < Bs) values were 300 and 350 °C for both steels (F (full transformation) treatments in Figure 1). The holding time, t_Iso_, was chosen according to previous experiences and to ensure that transformation is brought to its completion [19,20]. The times varied from 7 to 8 h for the 1C2Si treated at 350 and 300 °C, respectively, and the time was 1 h for the 04C3Si in all conditions. The austenitization and cooling rates to T_Iso_ were kept identical to those used in the Q experiment. An extra experiment for the 1C2Si treated at 250 °C for 14 h was also included to validate some of the procedures undertaken in this work. 

The volume fractions of retained austenite and bainitic ferrite at room temperature (RT) were determined from X-ray diffraction (XRD) patterns recorded in a Bruker D8 Advance diffractometer (Bruker AXS GmbH, Karlsruhe, Germany) equipped with a Goebel mirror and a LynxEye position sensitive detector (Bruker AXS GmbH, Karlsruhe, Germany). XRD spectra were collected in the coupled ϴ–2ϴ mode using Co X-ray tube working at 40 kV and 30 mA over a range from 10 to 120° with a step width of 0.01° and a counting time per step of 2 s.

Austenite fraction was calculated by comparing the areas under the ferrite peaks {110}, {200}, {211} and {220} with the areas under the austenite peaks {111}, {200}, {220} and {311}, assuming that the sample consists of only two phases [21]. In order to obtain more reliable and accurate results, the measured intensities of these diffraction peaks were corrected for preferred orientation using the cubic harmonic model [22]. The Rietveld refinement method [23] was applied to model the full pattern with the crystallographic information of ferrite and austenite by using version 4.2 of the program TOPAS (Bruker AXS). Besides global parameters such as background, zero displacement, scale factors and peak breadth, the refinement protocol also included the determination of the unit-cell parameters by the double Voigt approach [24]. For this analysis, the peak shape parameters of a corundum sample were used to remove the instrumental contribution to the width of the diffraction peaks. The austenite lattice parameter was used to calculate the austenite carbon content by using the formula proposed by Dyson and Holmes [25].

Samples used for XRD and microstructural observations were prepared following standard metallographic procedures that included grinding and polishing, finishing with 1 μm diamond paste. In samples used for XRD analysis, a final set of etching and polishing cycles were applied to remove the plastically deformed layer that may contain martensite induced by deformation introduced during the grinding step.

The different microstructures were revealed by etching with a 2% Nital solution for times ranging between 2 and 20 s, depending on the steel. Microstructures thus revealed were characterized using a field emission gun scanning electron microscope (FEG-SEM, JEOL Ltd, Tokyo, Japan). Bainitic ferrite plate thickness, t$\alpha_{b}$, was measured according to the procedure described in [26], which consists in measuring enough linear intercepts on SEM micrographs to obtain reliable statistics, i.e., at least 200 linear intercepts, and subsequently applying a stereographic correction to the mean linear intercept.

Vickers hardness with a 10 kg load was also measured, the presented results correspond to the average value of at least three measurements.

The needed thermodynamic calculations were performed using the free MUCG software from Cambridge University [27].

## 3. Results

### 3.1. Plate Thickness Evolution as Transformation Progresses

**Figure 3:**
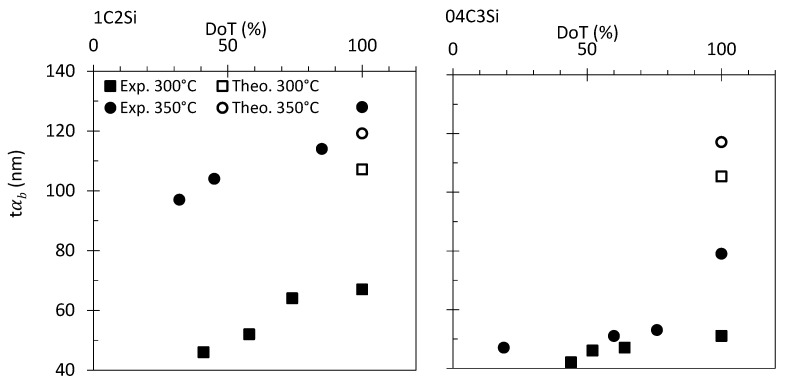
Bainitic ferrite plate thickness, *t*$\alpha_{b}$, as a function of the degree of transformation (DoT). Exp. and Theo. stand for experimental and theoretical values as described in the text.

To better understand the approach and structure of this work, we must first analyze the relevance of the results lying beneath its motivation, i.e., the evolution of the bainitic ferrite plate (${t\alpha }_{b}$) as a function of the degree of transformation (DoT) [Note that bainite transformation theory through the incomplete reaction phenomena, places a strict limit in how far the decomposition of austenite to bainitic ferrite can proceed before it becomes thermodynamically impossible for the transformation to proceed, i.e., incomplete reaction phenomena or $T_{0}^{}$ line concept [1]. This means that the transformation will stop well before full decomposition of the parent austenite from where it is forming. Therefore, in this work when the term DoT is used it refers to the percentage of transformation compared to that of the end of transformation (100%), and does not refer to the fraction of bainitic ferrite that has been formed] (see Figure 3). 

As mentioned, existing theory states that the most relevant factors contributing to the scale of the bainitic ferrite plates are the $YS_{\gamma }$ and ∆G [1,8,9,10,28], and the main outcome from that theory is that a strong austenite and high driving forces lead to a finer microstructure, the strength of the austenite being the most important factor. The bainite plate thickness, t$\alpha_{b}$, can be estimated by using the model proposed by Yang et al. [10], and the needed values of ∆G and $YS_{\gamma }$ can be estimated by means of MUCG software [27] and the Eres-Castellanos model [29], respectively. Hollow symbols in Figure 3 show the t$\alpha_{b}$ values for both steels at T_Iso_ 300 and 350 °C.

These calculations predict that for both steels, t$\alpha_{b}$ becomes thinner as T_Iso_ decreases, and for the same transformation temperature, the plate thickness of the 04C3Si alloy should be smaller than that of the 1C2Si steel, which is in accordance with the experimental values at DoT = 100% in Figure 3. What is puzzling and not predictable by the mentioned theory and model is that the plate thickness of bainitic ferrite becomes thicker as the transformation progresses (Figure 3).

During the transformation, at a given T_Iso_, there is a continuous variation of the values of both ∆G and $YS_{\gamma }$, as well as other parameters identified throughout the manuscript as relevant to the thickness of the resulting bainite plate. This should lead to a dependence of the bainite plate thickness on the DoT. 

The following sections will describe the procedures followed in order to characterize the evolution of the microstructure during transformation at T_Iso_ based on that at RT, which finally allow establishing certain relationships between the mentioned parameters and $t\alpha_{b}$ evolution as a function of the DoT.

### 3.2. Characterization of Bainitic Transformation and F and P Treatments; Microstructure and Transformation Characterization

**Figure 4:**
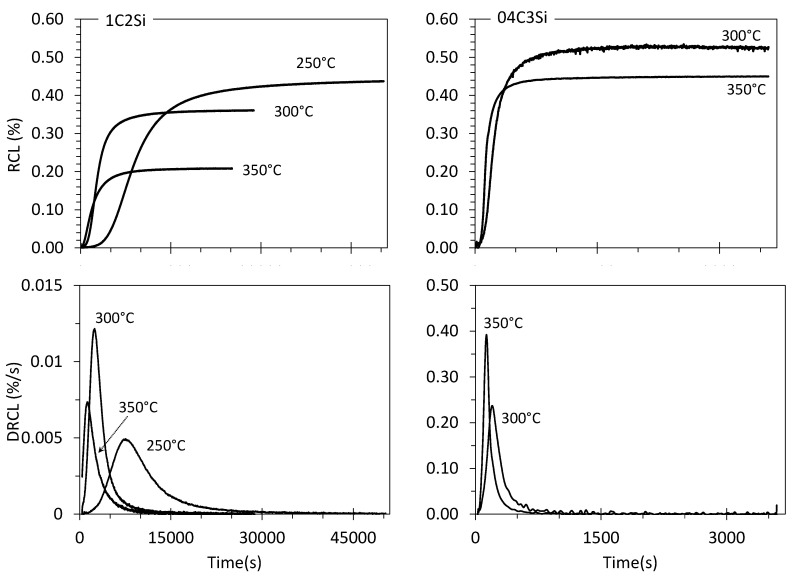
For both alloys, curves of the relative change in length (RCL) and its first derivative (DRCL) at different values of T_Iso_.

The evolution of the relative change in length (RCL) and its corresponding first derivative (DRCL) during the F treatment of Figure 1 are presented in Figure 4 for both steels at the selected T_Iso_. It should be mentioned that martensitic ($\alpha^{\prime }$) transformation was not detected on cooling to room temperature after the isothermal step in any of the tests. The RCL curves exhibit the typical sigmoidal shape, with a first part corresponding to the incubation period, where no transformation takes place or transformation is undetectable (RCL ~0). This incubation time decreases as T_Iso_ increases, ranging from 6 to 7 min and from ~1 to 1.5 min for the 1C2Si and 04C3Si steels, respectively. After this first period, the RCL curves show a characteristic steady increase associated with the continuous transformation of bainitic ferrite from the parent austenite. Finally, a steady state is reached (RLC approximately constant) when no more decomposition of austenite into ferrite takes place, regardless of the extension of the isothermal step. The RLC value at the plateau is directly related to the amount of bainitic ferrite formed, and, in agreement with bainitic transformation theory [1], its magnitude is higher the lower the T_Iso_ and the C content are [30,31,32]. 

A more careful analysis of the DRCL curves in Figure 4 provides further details on the evolution of the transformation. These curves show a very fast increase in the transformation rate followed by a maximum value (DRCL_max_) at the relatively early stages of the transformation, after which it becomes much slower. This DRCL_max_ is one order magnitude higher in the 04C3Si alloy as compared to the 1C2Si. 

**Figure 5:**
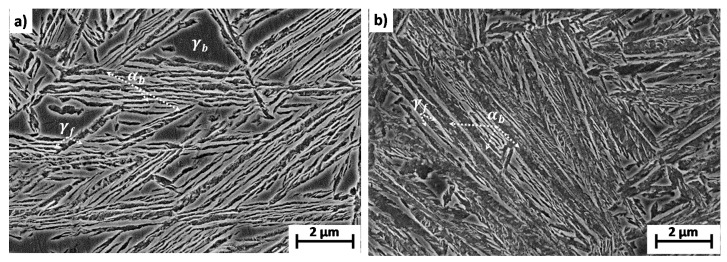
SEM micrographs of bainitic microstructures obtained after isothermal transformation at 300 °C (F test) in (**a**) 1C2Si steel and (**b**) 04C3Si steel, where $\alpha_{b}$ stands for bainitic ferrite and γ stands for retained austenite with film (f) and block (b) morphology.

From Figure 5, Table 2 and Table 3 (XRD (RT) F results), it is clear that regardless of the alloy and T_Iso_, the microstructure resulting from the described heat treatments consists of an interwoven mixture of bainitic ferrite (α_b_) plates with thin films of retained austenite ($\gamma_{f}$) and, trapped among the sheaves of bainite, some submicron blocks ($\gamma_{b}$) of retained austenite. As T_Iso_ decreases, there is a more abundant presence ($f\alpha_{b}$) of finer (t$\alpha_{b}$) and more C-saturated bainitic ferrite plates ($C\alpha_{b}$) (see Table 2 and Table 3 (XRD (RT) F results)), which results in harder microstructures. 

**Figure 6:**
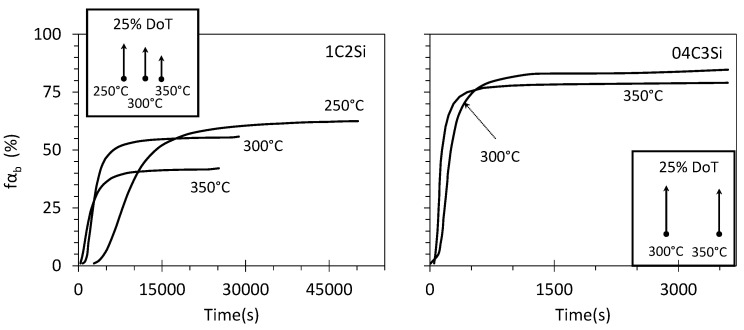
Evolution of the fraction of bainitic ferrite, $f\alpha_{b}$, as a function of time. Arrows correspond to the 25% degree of transformation (DoT) at the indicated T_Iso_.

As already mentioned, the relative change in length (RCL) accompanying bainitic transformation is directly related to the volume fraction of formed bainite ($f\alpha_{b}$); therefore, its evolution with time can be calculated from dilatation data using

(1) $${\left(f\alpha_{b}\right)}_{t,Iso}=\frac{{\left(RCL\right)}_{t}\;{\left(f\alpha_{b}\right)}_{F}}{{\left(RCL\right)}_{F}}$$

where the subindexes t and F indicate a given time and full transformation; i.e., ${\left(f\alpha_{b}\right)}_{F}$ corresponds to $f_{\alpha }$ F-values reported in Table 2 and Table 3. The results of such calculations are presented in Figure 6, where arrows that correspond to the 25% degree of transformation (DoT) at the indicated T_Iso_ are also presented.

Since no martensitic transformation was detected on cooling to RT, the fraction of austenite at a given T_Iso_ is directly calculated as

(2) $${\left(f\gamma \right)}_{t,Iso}=100-{\left(f\alpha_{b}\right)}_{t,Iso}$$

Based on the above-mentioned calculations, a set of interrupted experiments identified with P (partial) in Figure 1, Table 2 and Table 3 were performed using holding times, t_Iso_, selected within the region of steady increase in the RCL. In both tables, the expected fraction of bainitic ferrite, $f\alpha_{b,Iso}$, and the degree of transformation (DoT) correspond to those obtained using Equation (1) and Figure 6, following the procedures just described.

**Figure 7:**
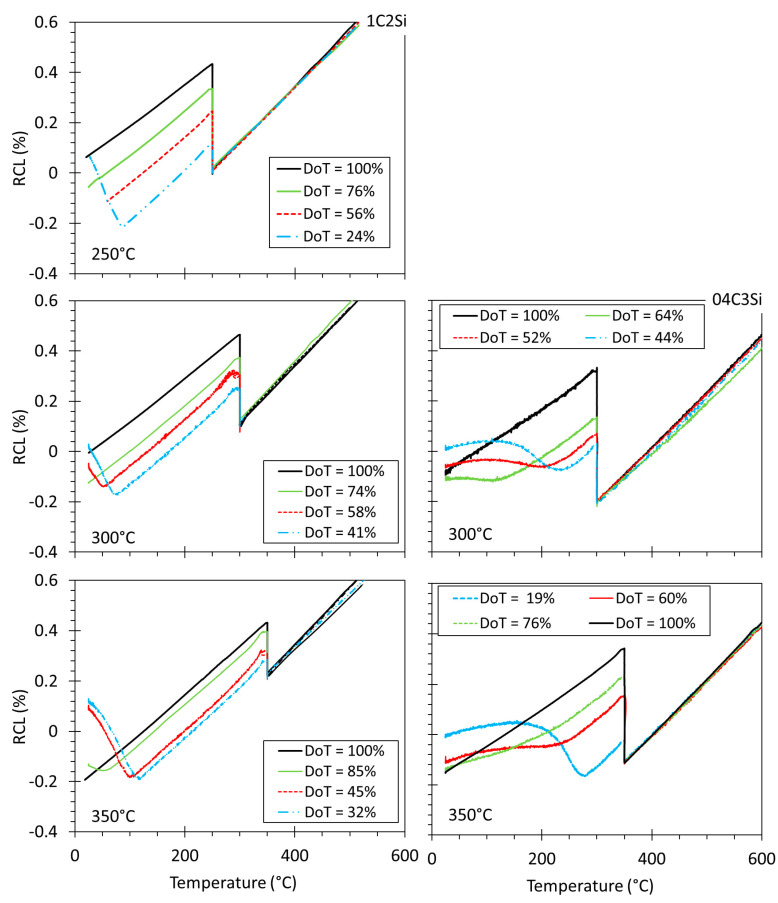
Relative change in length (RCL) of partially (P) and fully (F) transformed experiments; the degree of transformation (DoT) is also indicated.

**Figure 8:**
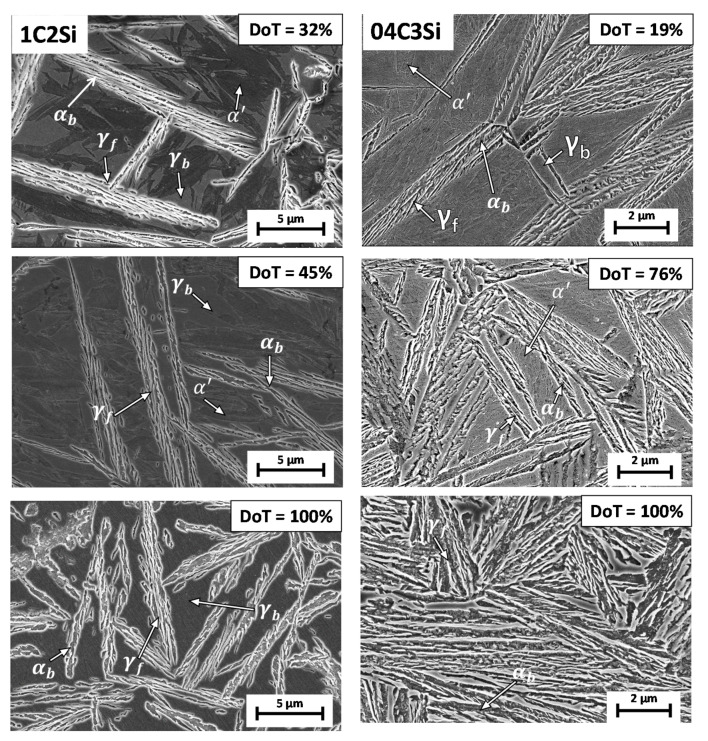
Examples of the evolution of the 350 °C microstructure for both alloys. DoT stands for the degree of transformation, $\alpha_{b}$ stands for bainitic ferrite, $\alpha^{\prime }$ stands for martensite and γ stands for retained austenite with film (f) and block (b) morphology.

Figure 7 shows that the level of reproducibility of the different P tests is good but also that the level of C enrichment in austenite (due to bainitic transformation) is not always sufficient to stabilize such phase down to room T. In the case of the 1C2Si steel at 250 °C, the lowest T_Iso_, martensite is detected only when the degree of bainitic transformation is 25%, while at higher T_Iso_, a greater degree of transformation (DoT) is needed to retain austenite (see Table 2). The situation is even more pronounced in the 04C3Si steel, where it is clear that only the 100% transformation (F tests) ensures sufficient C enrichment in austenite to avoid martensitic (α’) transformation on cooling (see Table 3). Selected illustrative examples of the obtained microstructures are presented in Figure 8. 

Therefore, the measured Ms temperatures shown in Figure 7 (see Table 2 and Table 3) tend to decrease as the degree of bainitic transformation increases, as does the martensite fraction $f\alpha^{\prime }$, calculated as

(3) $$f\alpha^{\prime }=100-{\left(f\gamma \right)}_{RT}-f\alpha_{b}$$

When comparing the $f\alpha_{b,Iso}$ values calculated by means of Equation (1) (Table 2 and Table 3) and those determined from the XRD patterns, it can be concluded that they agree quite well only when no fresh martensite $\alpha^{\prime }$ is formed during cooling to room temperature (RT), which validates the approach undertaken with Equation (1).

The carbon content of martensite, $C\alpha^{\prime }$, was obtained using the experimental Ms and the expression of Payson and Savage [15]. Note that the mentioned expression was deemed the most appropriate for these chemical compositions given the excellent agreement between experimental and calculated Ms values for the Q experiments in Table 1. 

As is clear from the XRD results in Table 2, where no $\alpha^{\prime }$ was detected at RT, C within bainitic ferrite, $C\alpha_{b}$, remains constant through the whole transformation, results that are in line with those previously reported for this type of microstructure [33]. Therefore, it is safe to assume that the calculated $C\alpha_{b}$ value for the F experiments is also that for all the P experiments where it was not possible to separate in the XRD patterns the contribution of both fresh martensite and bainitic ferrite.

With all this information, the only remaining parameter needed to have a full picture of the evolution of the microstructure at the transformation T_Iso_ is the C content of austenite, ${\left(C\gamma \right)}_{Iso}$, which can be calculated using the following lever rule, which takes into account the fraction of austenite that transformed to $\alpha^{\prime }$ on cooling to RT and the values of fγ and Cγ obtained from the Rietveld refinement performed on the XRD patterns recorded at room temperature:

(4) $${\left(C\gamma *f\gamma \right)}_{Iso}={\left(C\gamma *f\gamma \right)}_{RT}+{\left(C\alpha^{\prime }*f\alpha^{\prime }\right)}_{RT}$$

where $f\gamma_{Iso}$ was obtained by Equation (2) and $C\alpha^{\prime }\;\mathrm{and}\;f\alpha^{\prime }$ were obtained as described in the previous paragraphs.

**Figure 9:**
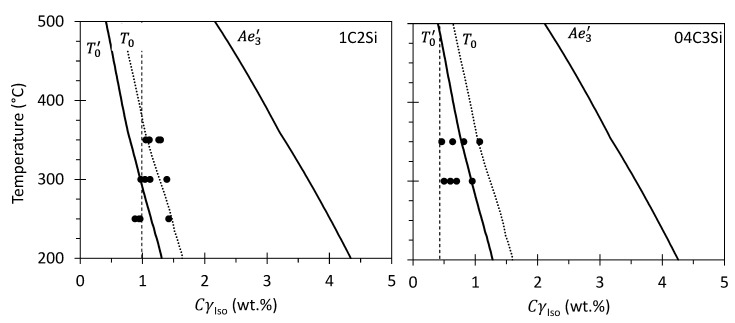
A comparison of determined carbon content of austenite at different isothermal T ($C\gamma_{Iso}$) values, solid circles, against calculated $T_{0}^{}$, $T_{0}^{\prime }$ and $Ae_{3}^{\prime }$ phase boundaries. Vertical dashed line represents the average C content of the alloy.

The resulting $C\gamma_{Iso}$ values (Table 2 and Table 3 and plotted in Figure 9), together with the $T_{0}^{\prime }$ line and the paraequilibrium γ+α/γ phase boundaries $Ae_{3}^{\prime }$ [27], show that the obtained results complied with the incomplete reaction phenomena that rule bainitic transformation [1]. This means that as the transformation progresses, the carbon concentration of austenite increases, and at the point where the reaction terminates its value is far less than demanded by equilibrium $Ae_{3}^{\prime }$ and close to that expected from the $T_{0}^{\prime }$ phase boundary after allowing for the heterogeneous distribution of C. According to this theory, the $T_{0}^{}$ temperature in steel is defined as that at which ferrite and austenite of the same chemical composition have identical free energy, if the strain energy due to the displacements associated with the transformation is considered, and then is defined as the $T_{0}^{\prime }$ temperature. The same theory demonstrates that it is thermodynamically impossible for the austenite to transform to ferrite of the same chemical composition once it reaches the C concentration given by the $T_{0}^{\prime }$. It has to be noted that in the microstructures where martensite is present, compression stresses are introduced in the surrounding regions (austenite) caused by the displacive character of the transformation, which in turn means that the $C\gamma_{RT}$ value and consequently also $C\gamma_{Iso}$ are underestimated [34,35,36]. This explains that at low DoT, where higher fractions of $\alpha^{\prime }$ are present, $C\gamma_{Iso}$ is very close to, or may be even less than, the average fraction of carbon present in the alloy (dashed vertical line in Figure 9).

### 3.3. Rationalization of the Dynamic Character of the System; Understanding the Evolution of Bainitic Ferrite Plate Thickness as Transformation Progress

Carbon diffusion from supersaturated bainitic ferrite into the remaining austenite right after a plate of bainite stops growing has several effects. On the one hand, the continuous increase in $C\gamma_{Iso}$ as transformation advances chemically stabilizes this phase and hinders further transformation. On the other hand, as C is a strong solid solution strengthener, it increases the $YS_{\gamma }$ which also opposes the transformation. What is more, bainite formation involves the increase in the dislocation density of the parent austenite, which has two contrary effects on transformation kinetics. First, the higher dislocation density increases the nucleation site density for phase transformation, which potentially accelerates the transformation kinetics. Second, in addition to the increasing $YS_{\gamma }$, the accommodation of the plastic deformation by the displacive character of the transformation is hindered at higher dislocation densities, which affects the driving force for growth by increasing the elastic energy in austenite, i.e., mechanical stabilization of austenite [37]. In other words, the system is in continuous evolution as the transformation proceeds, and the associated changes in chemical composition and dislocation density have a hindering effect on the transformation kinetics. This sluggishness in the advancing transformation is reflected by the reduction in the transformation rates once the DRCL_max_ maximum is achieved, and for the present alloys, such a maximum has been calculated to occur at DoT of ~40% and ~43–57% for the 1C2Si and 04C3Si, respectively (see Figure 4). 

**Figure 10:**
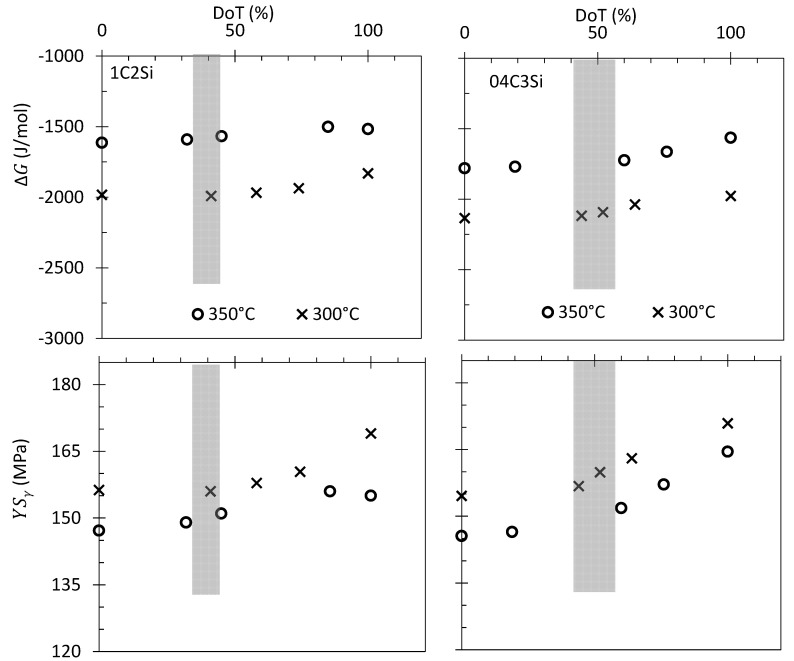
Variation of both the driving force for bainitic transformation (∆G) [27] and the $YS_{\gamma }$ [29] as a function of the degree of transformation (DoT). The shaded regions represent where the DRCL_max_ is reached.

To illustrate these assumptions, the driving force for the transformation (∆G) of austenite into bainite [27] and its $YS_{\gamma }$ [29] values have been recalculated as a function of their corresponding $C\gamma_{Iso}$ and represented as a function of the DoT in Figure 10. The same image contains shaded areas representing where the DRCL_max_ is achieved. It becomes evident that there is a decrease in the available driving force for the transformation as it proceeds, which is also accompanied by an important increase in the $YS_{\gamma }$ value, being more pronounced after the DRCL_max_ (shaded regions), providing some evidence to explain the observed deceleration of the transformation, and in line with recent results [37].

**Figure 11:**
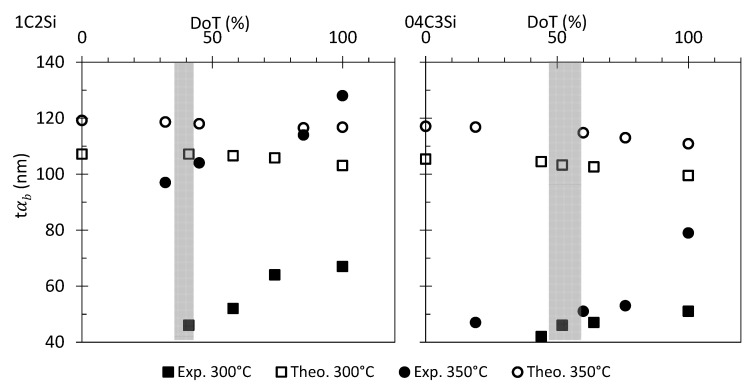
Bainitic ferrite plate thickness, t$\alpha_{b}$, as a function of the degree of transformation (DoT). Exp. and Theo. stand for experimental and theoretical values as described in the text. The shaded regions represent where the DRCL_max_ is reached.

For both steels, treated at the same temperatures of 300 and 350 °C, the plate thickness (t$\alpha_{b}$) has been measured at the different DoTs (see Table 2 and Table 3 and Figure 11). The same figure contains the t$\alpha_{b}$ values calculated as in [10], considering the corresponding values of $YS_{\gamma }$ and ∆G at each of the studied DoTs. While calculations are able to anticipate the experimentally observed decrease in ${t\alpha }_{b}$ as T_Iso_ is lowered, as well as the refinement of the 04C3Si microstructures as compared to those of the 1C2Si steel, it predicts that a refinement of the microstructure should be expected as transformation proceeds, contrary to the experimental evidence that shows an increase in the plate size (Figure 11). 

**Figure 12:**
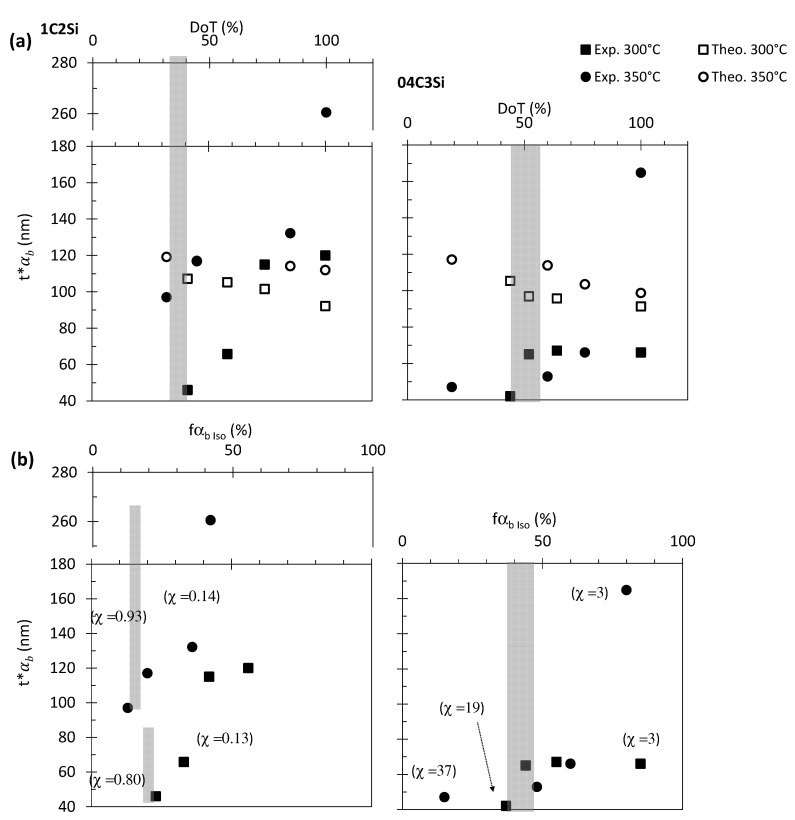
Bainitic ferrite plate thickness, t*$\alpha_{b}$, as a function of (**a**) the degree of transformation (DoT) and (**b**) fraction of bainitic ferrite,$\;f\alpha_{b,Iso}$. The shaded regions represent where the DRCL_max_ is reached.

Note that presented $t\alpha_{b}$ values correspond to the average measured in the microstructure at a given DoT and T_Iso_, following Equation (5).

(5) $$t\alpha_{b}=\overset{DoT=\ldots ,100\%}{\underset{DoT=initial}{\sum }}{\left(f\alpha_{b}\right)}_{DoT}{\left(t^{*}\alpha_{b}\right)}_{DoT}/\overset{DoT=\ldots ,100\%}{\underset{DoT=initial}{\sum }}{\left(f\alpha_{b}\right)}_{DoT}$$

where ${\left(t^{*}\alpha_{b}\right)}_{DoT}$ and ${\left(f\alpha_{b}\right)}_{DoT}$ correspond to the actual plate thickness and fraction of bainitic ferrite formed at each stage, i.e., DoT, considering the fraction and sizes that formed in previous stages. Thus, the values of the ${\left(t^{*}\alpha_{b}\right)}_{DoT}$ should better describe the plate thickness behavior as the transformation proceeds, and they are presented in Figure 12a for both the theoretical and experimental measurements. As seen, the increase in the size of the plates formed at each of the DoTs where it was measured becomes more evident. The discrepancy between the theoretical and experimental tendencies evidences that, in addition to $YS_{\gamma }$ and ∆G, other contributions need to be considered as relevant to the control of the bainite ferrite plate thickness.

The thickness must also be influenced by impingement between adjacent plates. As in all transformations, a larger transformation rate corresponds to a finer microstructure. Chang et al. reported that the thickness of a plate can increase even after lengthening has halted until the chemical force is exhausted by the accumulation of strain energy, thermoelastic equilibrium, or by the presence of adjacent parallel plates, hard impingement [28]. Therefore, it can be considered that, at the early stages of the transformation, when the transformation rate is evolving to its maximum, a high fraction of bainitic ferrite plates form and grow, and the chances of hard impingement are higher. As the DRCL_max_ is overcome and the transformation becomes more sluggish, lower transformation rates make hard impingement events less likely to occur, allowing for the thickening of the plates. 

The previous assumptions may be supported by defining χ, as the amount of bainitic ferrite fraction ($f\alpha_{b}{}_{Iso}$) transformed per minute, at two different stages of the transformation, namely (i) from the start of the transformation to the point where the maximum rate is achieved (DRCL_max_) and (ii) from that point to the end of the transformation, and by representing the evolution of $t^{*}\alpha_{b}$ as a function of $f\alpha_{b}{}_{Iso}$ (see Figure 12b). 

The first thing to note is the order of magnitude difference in the χ values of 04C3Si as compared to those of the 1C2Si, which reflects what has been already described: a much bigger fraction of bainitic ferrite is achieved in shorter times (see Figure 6). This must translate into a higher probability of hard impingement events. In addition, a high transformation rate is equivalent to a high strain rate, which increases $YS_{\gamma }$ and therefore also limits the thickness of the plates. To a certain extent, these results would explain why, despite the similar $YS_{\gamma }$ and ∆G values at a given T_Iso_ for both steels, the plates tend to be much thinner in the 04C3Si alloy (see Figure 11). It is also noticeable how, for both steels, the value of χ up to the DRCL_max_ is bigger than that after this maximum. Thus, the biggest increase in plate thickness is always achieved after the DRCL_max_ (shaded area in Figure 12b), when the rate of transformation is at its lowest; i.e., it is less likely that hard impingement events occur as opposed to the situation before the DRCL_max_ is reached.

It should not be forgotten that in the absence of hard impingement obstacles, a plate would thicken until the thermoelastic equilibrium occurs. Being a thermally activated process, such an effect is more likely to occur at higher T_Iso_, which could also help to understand the important increase in the value of $t^{*}\alpha_{b}$ detected for both steels at DoT = 100% and T_Iso_ = 350 °C (Figure 12b), where the transformation rate is almost exhausted with almost no hard impingement events occurring.

There is also the fact that, due to its displacive character, as the transformation progresses, there is an increase in the dislocation density needed for the austenite to accommodate the plastic deformation inherent to the formation of the bainitic plates. The multiplication of dislocations during strain accommodation repels the further thickening of bainitic ferrite plates [1,12]. The contribution of dislocations must undoubtedly be considered, but in view of the experimental results presented, this contribution does not seem to be as important a parameter as the kinetics of transformation and the associated impingement events. 

## 4. Conclusions

The adopted approach in this work allowed obtaining a dynamic picture of the bainitic transformation by means of the detailed characterization of the resulting microstructure at room temperature. It has been shown that as the transformation proceeds, there is an increase in the YS of the austenite from where bainite grows and a decrease in the driving force for the transformation. Those changes become more evident after the characteristic maximum of transformation rate, which occurs at the early stages of the transformation and leads to a stage where there is a significant deceleration of the transformation up to its end. It is that same change in the kinetics of the transformation that has been found to have a stronger influence on the bainite ferrite plate thickness than anticipated by existing theories, which attribute to the austenite YS and to the driving force of the transformation the strongest influence in controlling the final scale of bainitic ferrite. 

Experiments have shown that the plate thickness tends to increase as the transformation proceeds, and the effect is more pronounced once the transformation enters the mentioned deceleration stage. Such behavior, opposite to that anticipated by the existing theory and models, has been found to have a strong correlation with the mentioned rate of transformation and most likely with the concomitant hard impingement events.

Therefore, more work is needed in order to incorporate into new or existing models (i) all the parameters that have been identified as relevant in the definition of the final scale of the bainitic ferrite and (ii) the dynamic character that those parameters have in the sense that they change as the transformation advances.

## Figures and Tables

**Table 1:** Chemical composition of the steels and experimental critical temperatures obtained from dilatometric curves as described in the main body of the text. Theoretical Ms and Bs calculated according to provided references.

Alloy	Composition (wt.%)						T (°C)				
	C	Si	Mn	Cr	Mo	Cu	Ac1	Ac3/Acm	Ms	Ms [15]	Bs [16]
**1C2Si**	0.99	2.4	0.75	0.98	0.02	0.19	815	844	112	106	425
**04C3Si**	0.43	3.05	0.71	0.97	0.21	0.14	845	915	287	276	566

**Table 2:** Volume fraction (fi) and carbon content (Ci) of identified phase in the microstructures of 1C2Si at room temperature; $\alpha_{b}$ is bainite, $\alpha^{\prime }$ is martensite, γ is austenite and $\alpha =\alpha_{b}+\alpha^{\prime }$. P and F stand for partial and full transformation, while RT refers to room temperature and Iso refers to isothermal temperature. The martensitic transformation start temperature Ms, plate thickness (*t*$\alpha_{b}$) and hardness HV10 are also included. An asterisk (*) indicates that no martensite was detected on cooling to room T. Dil. stands for dilatometry and DoT stands for degree of transformation.

T_Iso_	Dil.		XRD (RT)				Dil. (Iso)		RT	Iso	Dil.		
		**DoT (%)**	fγ (%) **(±3)**	$C\gamma_{RT}\;(\mathrm{wt}.\%)$ **(±0.12)**	$f_{\alpha }\;(\%)$ (±3)	$C\alpha_{b}\;(\mathrm{wt}.\%)$ (±0.03)	$f\alpha_{b,\;Iso}\;(\%)$ Equation (1)	$f\alpha^{\prime }\;(\%)$ Equation (3)	$C\alpha^{\prime }\;(\mathrm{wt}.\%)$ Ref. [15]	$C\gamma_{Iso}\;(\mathrm{wt}.\%)$ Equation (4)	Ms (°C)	$t\alpha_{b}\;(\mathrm{nm})$ (±10) [26]	**HV10**
250 °C	P	24	40	0.78	60	–	16	44	1.1	0.95	83		654 ± 4
		56 *	63	0.88	37	0.19	34	–	–	0.88	–		567 ± 1
		76 *	55	0.96	45	0.18	48	–	–	0.96	–		575 ± 3
	F	100 *	37	1.42	63	0.19	=	--	--	1.42	--		635 ± 2
300 °C	P	41	53	0.90	47	–	23	24	1.13	0.97	73	46	604 ± 2
		58	54	1.01	46	–	33	13	1.19	1.04	53	52	577 ± 5
		74 *	55	1.12	45	0.19	42	–	–	1.12	–	64	516 ± 1
	F	100 *	44	1.39	56	0.20	=	–	–	1.39	–	67	515 ± 7
350 °C	P	32	41	1.10	59	–	13	46	1.01	1.05	110	97	690 ± 5
		45	43	1.16	57	–	20	37	1.05	1.11	98	104	611 ± 5
		85	58	1.30	42	–	36	6	1.19	1.29	52	114	475 ± 6
	F	100 *	58	1.26	42	0.16	=	–	–	1.26	–	128	383 ± 1

**Table 3:** Volume fraction (fi) and carbon content (Ci) of identified phase in the microstructures of 04C3Si at room temperature; $\alpha_{b}$ is bainite, $\alpha^{\prime }$ is martensite, γ is austenite and $\alpha =\alpha_{b}+\alpha^{\prime }$. P and F stand for partial and full transformation, while RT refers to room temperature and Iso refers to isothermal temperature. The martensitic transformation start temperature Ms, plate thickness (*t*$\alpha_{b}$) and hardness HV10 are also included. An asterisk (*) indicates that no martensite was detected on cooling to room T. Dil. stands for dilatometry and DoT stands for degree of transformation.

**T_Iso_**	**Dil.**		XRD (RT)				Dil. (Iso)		RT	Iso	Dil.		
		**DoT (%)**	fγ (%) **(±3)**	$C\gamma_{RT}\;(\mathrm{wt}.\%)$ **(±0.12)**	$f_{\alpha }\;(\%)$ (±3)	$C\alpha_{b}\;(\mathrm{wt}.\%)$(±0.03)	$f\alpha_{b,\;Iso}\;(\%)$ Equation (1)	$f\alpha^{\prime }\;(\%)$ Equation (3)	$C\alpha^{\prime }\;(\mathrm{wt}.\%)$ Ref. [15]	$C\gamma_{Iso}\;(\mathrm{wt}.\%)$ Equation (4)	Ms (°C)	$t\alpha_{b}\;(\mathrm{nm})$ (±10) [26]	**HV10**
300 °C	P	44	10	0.37	90	–	37	53	0.52	0.5	254	42	674 ± 1
		52	9	0.28	91	–	44	47	0.64	0.6	213	46	660 ± 3
		64	15	0.56	85	–	55	30	0.88	0.7	140	47	616 ± 2
	F	100 *	15	0.95	85	0.19	=	–	–	0.95	–	51	578 ± 4
350 °C	P	19	5	0.63	95	–	15	80	0.45	0.46	276	47	702 ± 5
		60	10	0.95	90	–	48	42	0.56	0.64	242	51	609 ± 9
		76	15	1.04	85	–	60	25	0.68	0.82	205	53	533 ± 3
	F	100 *	20	1.07	80	0.16	=	–	–	1.07	–	79	502 ± 0

## Data Availability

The raw/processed data required to reproduce these findings cannot be shared at this time as the data also forms part of an ongoing study.
